# Identification of Novel Pesticides for Use against Glasshouse Invertebrate Pests in UK Tomatoes and Peppers

**DOI:** 10.3390/insects6020464

**Published:** 2015-05-26

**Authors:** David R. George, Jennifer A. Banfield-Zanin, Rosemary Collier, Jerry Cross, A. Nicholas E. Birch, Roma Gwynn, Tim O’Neill

**Affiliations:** 1 Stockbridge Technology Centre, North Yorkshire YO8 3TZ, UK; E-Mail: jen.banfield-zanin@stc-nyorks.co.uk; 2 Department of Applied Sciences, Northumbria University, Newcastle Upon Tyne NE1 8ST, UK; 3 Warwick Crop Centre, Warwick CV35 9EF, UK; E-Mail: Rosemary.Collier@warwick.ac.uk; 4 East Malling Research, Kent ME19 6BJ, UK; E-Mail: Jerry.Cross@emr.ac.uk; 5 James Hutton Institute, Dundee DD2 5DA, UK; E-Mail: Nick.Birch@hutton.ac.uk; 6 Rationale, Duns TD11 3QA, UK; E-Mail: rgwynn@biorationale.co.uk; 7 ADAS, Cambridge CB23 4NN, UK; E-Mail: Tim.O'Neill@adas.co.uk

**Keywords:** pesticide, thrips, whitefly, two-spotted spider mite, aphid, tomato, pepper, SCEPTRE project

## Abstract

To inform current and future pesticide availability to glasshouse vegetable growers, the current project trialled more than twenty products, including existing industry standards, against four key pests of glasshouse tomatoes and bell peppers. These included experimental conventional chemical pesticides as well as alternative biopesticide and biorational products based on phytochemicals, microbials and physically-acting substances. The results suggest that certain biopesticide products, particularly botanicals, provide good levels of pest control, with the same being true of experimental conventional chemical pesticides not yet recommended for use against these pests on these crops. Efforts are on-going to ensure that results of the current project translate to industry benefit via new pesticide approvals.

## 1. Introduction

In the UK, some 818 hectares are devoted to the growing of protected vegetables. Tomato is one of the most important crops in terms of both value and area cropped, and is classified as a major crop by Defra (the Department for Environment, Food and Rural Affairs). Over 200 hectares of glasshouse area are used to produce British tomatoes and the total tonnage of fresh tomatoes consumed in the UK is increasing. The value per planted hectare of tomato is estimated at £474,000 [1], with the retail value of British tomato production around £175 million. Bell peppers, by contrast, are considered a minor crop, and take up some 85 hectares of UK glasshouse area. Despite this, they are a high value crop, with an estimated worth over £200,000 per planted hectare [1].

Glasshouse production has many benefits, not least that it allows production of crops such as tomato and pepper under temperate climates in countries, including the UK. In addition, glasshouse systems may afford some level of protection from pests, limiting access to the crop through creation of a physical barrier to inward pest movement that may be fortified through screening with appropriate mesh sizes [2]. Even with screening in place, however, pest penetration is often significant [3] and supplementary control measures are typically required [4]. Indeed, the artificial system created, with controlled and buffered temperature and humidity and without wind and rain, exacerbates pest problems where they do occur by providing relatively optimal conditions for pest population growth [3,5].

Where pest outbreaks occur on protected edible crops in Europe, conventional chemical pesticides are still widely utilised in their control, the cost of which exceeds (US)$1 billion for two-spotted spider mite (*Tetranychus urticae*) alone, albeit across multiple sectors [6]. Nevertheless, reliance on biological control of pests through release of pest natural enemies is commonplace in glasshouse production systems worldwide [7], having been developed since the 1960s to provide in excess of 100 commercially-available biocontrol species today [5]. However, despite these advances in biocontrol of glasshouse pests, aphid, thrips, spider mite and whitefly populations in particular remain an ever-present threat, especially where resistance to conventional chemical pesticides has been documented (e.g., [8,9]). Though biological control may be effective against these pests, results may be variable and dependent upon factors such as release timings, grower experience and incidence of hyper-parasitism/predation, which can lead to biocontrol failure [10]. Where biocontrol does break down, growers often turn to conventional chemical pesticides to curtail pest populations, further disrupting biological or integrated pest management (IPM) programmes (D. George, personal observation). Consequently, the predicted and complete phasing-out of conventional chemical pesticides from the glasshouse sector by 2010 has not been realised as some predicted [5], at least in the majority of cases. In contrast, there remains a need to identify new products to target pest outbreaks under glass. Biopesticides are likely to be particularly important here; as future use of numerous conventional chemical pesticides is threatened in the UK and Europe [11], alternative products are likely to become ever more utilised in crop production. The Compound Annual Growth Rate of biopesticides (16%) already far exceeds that of conventional chemical pesticides, albeit from a much smaller base, with the global market for biopesticides (including fungicides, herbicides and nematicides) expected to reach (US)$4.4 billion by 2019, from (US)$1.8 billion in 2013 [12].

Benefits of biopesticides (semiochemicals, botanicals and microbials) and biorationals (e.g., physical pest disruptors such as fatty acids), are reported as the potential for reduced occurrence of pest resistance [13,14], higher amenability to combined use with biological control [15,16], and lowered environmental persistence with reduced product residues [13,17,18]. Such products also align better with modern consumer/retailer demands, with many, though not all, meeting organic production standards [19]. Biopesticide and biorational (henceforth grouped as “biopesticide”) products are not without issue, however, typically achieving lower and slower overall kill rates than conventional chemical pesticides [20], being more sensitive to environmental degradation [14], and often having a requirement for direct pest contact that necessitates high spray volumes and more informed application procedures [19]. Thorough testing of both “new” conventional chemical and biopesticide products against industry standard regimes is therefore critical to identify those products with the greatest potential to contribute to future management of glasshouse pests. This is especially true in the UK where the approved number of biopesticides is relatively small in comparison to countries such as the Netherlands [21].

With the above in mind, the aim of the current project was to assess the efficacy of a number of new experimental conventional chemical and biopesticide products against selected glasshouse pests of tomato and bell pepper in order to provide data to support new product approvals for the protected edibles sector. Products and target pests were selected through industry consultation, and all products tested against industry standard pesticides and negative controls.

## 2. Experimental Section

From the outset of the project, a number of key priority target pests were identified following discussion with the “Protected Crops Panel” of the Horticultural Development Company, individual growers and crop consultants. Two glasshouse crops were selected for study, tomato (*Solanum lycopersicum*, var. Dometica) and bell pepper (*Capsicum annuum*, var. Ferrari), with experiments conducted on five key pests of these crops: western flower thrips (*Frankliniella occidentalis* (Pergande)), glasshouse whitefly (*Trialuerodes vaporariorum* (Westwood)), two-spotted spider mite (*Tetranychus urticae* (Koch)) and the glasshouse-potato and peach-potato aphids (*Aulacorthum solani* (Kaltenbach) and *Myzus persicae* (Sulzer), respectively).

**Figure 1:**
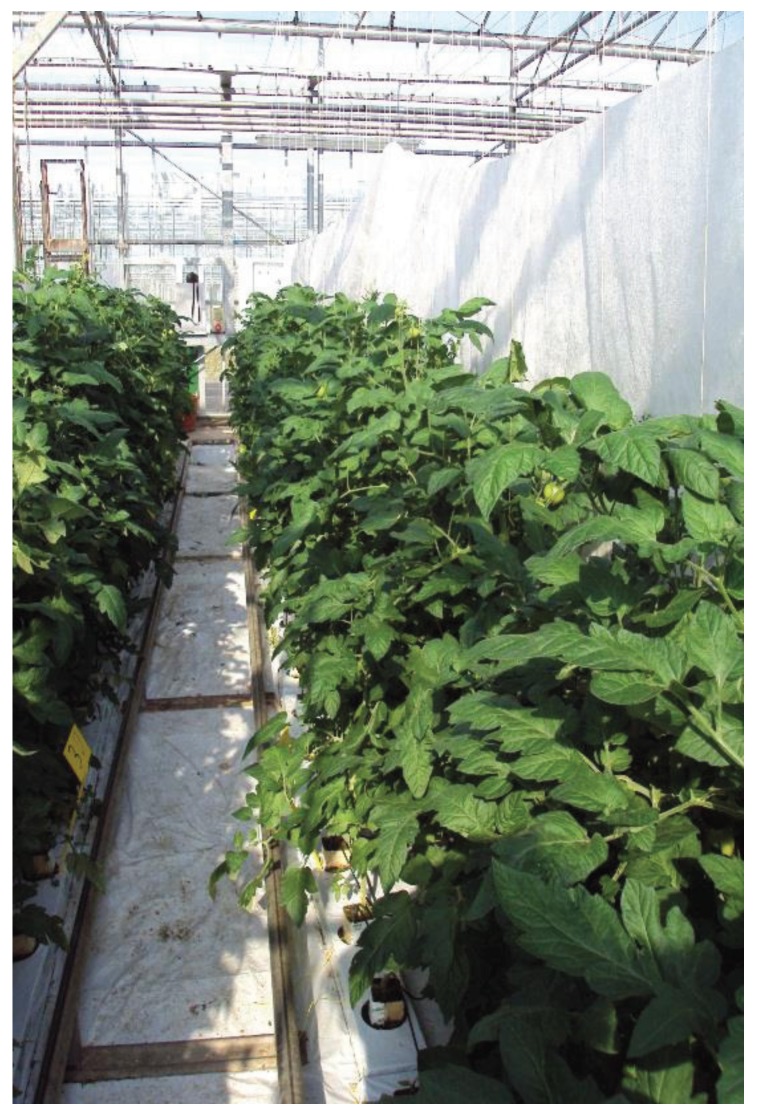
Tomato crop showing rockwool blocks, slabs and path/fleece divides.

All trials utilised plants grown in rockwool blocks under glasshouse conditions at Stockbridge Technology Centre, North Yorkshire, UK (Figure 1). Plants were transferred to rockwool slabs (three plants per slab) when sufficiently mature and thereafter maintained according to standard commercial practises, with the exception of pest control measures. Treatments were arranged in a randomised block design, utilising two rockwool slabs as an experimental plot. Plots were always separated by at least one rockwool slab to serve as a “guard” and limit pest movement between plots. Guard blocks were also positioned at the ends of any row of plants to limit edge effects, with rows adjacent to glasshouse walls being made up entirely of guard plants for the same reason. Spaces between rows consisted either of concrete pathways or fleece partitions (Figure 1), erected to prevent spray drift and limit pest movement between plots. Plants were always transferred to glasshouses at commercially applicable densities, with six replicates of each treatment, including an industry standard pesticide, plus a water-only control, always included.

**Figure 2:**
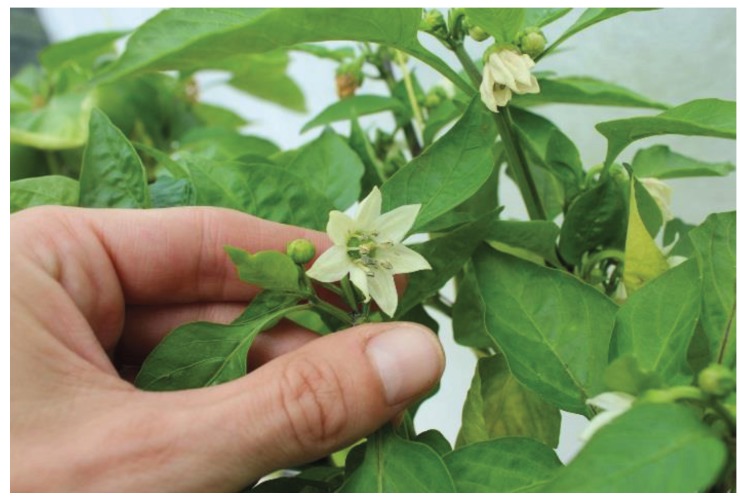
Pepper flower head prior to excision with *Frankliniella occidentalis* visible.

**Figure 3:**
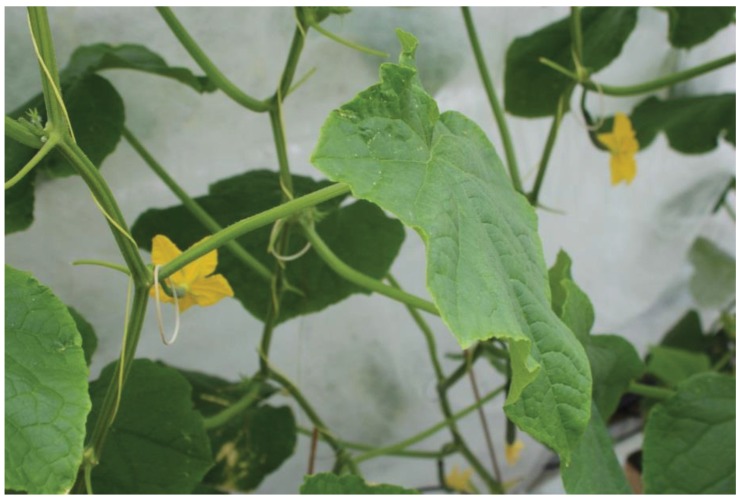
Tomato leaf showing signs of *Tetranychus urticae* feeding damage and rubber bands used to mark leaves on which mites were released.

**Figure 4:**
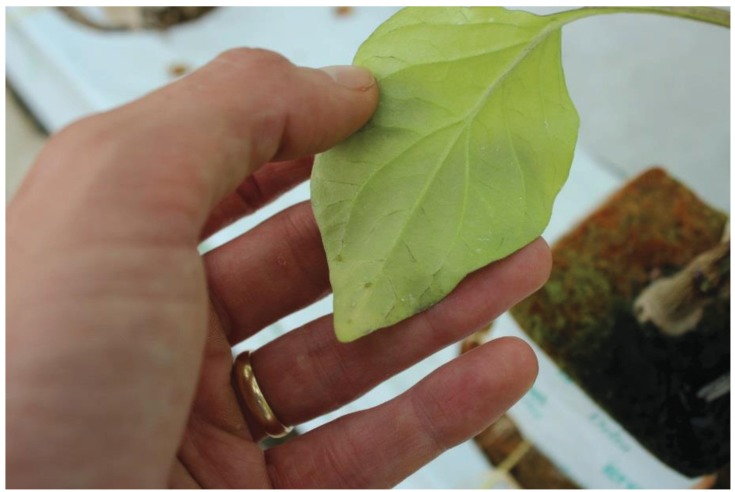
Pepper leaf with *Aulacorthum solani* on abaxial surface (NB: aphids were recorded from all plant parts).

Once transferred to glasshouses, plants were infested with pests that had been reared in either an insect growth room or bio-houses (small glasshouses) present at the trial site. Pests were transferred to crop plants either by releasing adult insects into plots (*T. vaporariorum* only), or by inoculation with infested plant material (all other pests). Once infested, pests were given a short period to settle before pre-treatment counts of pest numbers (at date “D0”) were made according to guidelines provided by the European Plant Protection Organisation, which were consulted at all stages of trial design. Counts of *T. vaporariorum* were made from top, middle and lower leaves of each plant in a plot, recording adults and larvae (all stages) separately; counts of *F. occidentalis* were made from two excised flower heads per plant (Figure 2), for each plant in a plot, also counting adults and nymphs separately. Counts of *T. urticae* (adults and nymphs) were made from each plant in a plot, from marked leaves on which mites had been previously released (Figure 3), and counts of aphids (all stages combined) were made from whole plants on each plant in a plot, taking care to examine both adaxial and abaxial leaf surfaces (Figure 4). Where pre-treatment counts were deemed too low to commence treatment, the infestation process was repeated.

Once sufficient pest levels had been reached, plots were treated with pesticide products at concentrations recommended by manufacturers, deployed in water such that all plots in a given experiment received the same overall spray volume (750 L/Ha/mch). Each treatment was applied by qualified personnel using a single-lance Oxford Precision Sprayer, fitted with a hollow-cone nozzle, at approximately 3 bar pressure. Due to the nature of the biopesticides being tested, care was taken to ensure complete plant coverage and thorough mixing of products (in water) prior to and during treatment delivery, with pest counts made six days after each application (6DAA). The timing and number of applications for some pesticides varied within trials to conform to existing or anticipated regulatory requirements and product labelling. This was particularly so for industry standard pesticides, which were often applied on a reduced number of occasions to conform to existing constraints on their use (see Table 1), as informed by product information held in the LIAISON pesticide database <<liaison.fera.defra.gov.uk/>>.

**Table 1:** Target pests, crops and treatments for glasshouse pest trials conducted between 2012 and 2014. Experimental treatments (*i.e.*, products) are coded for confidentiality, though a distinction is made between experimental conventional chemical pesticides (codes starting with “C”) and biopesticides: B = botanical; M = microbial; P = physically-acting. Control treatments were always water-only and industry standards are denoted first by trade name, then by the primary active ingredient.

YEAR	2012		2013	2014	
CROP	Tomato	Tomato	Pepper	Pepper	
**PEST**	*T. vaporariorum*	*T. urticae*	*M. persicae*	*F. occidentalis*	*A. solani*
**PRODUCTS TESTED**	Control	Control	Control	Control	Control
	Chess WG ^3^ (pymetrozine)	Borneo ^1^ (etoxazole)	Pyrethrum 5EC (pyrethrin)	Calypso ^2^ (thiacloprid)	Chess WG ^3^ (pymetrozine)
	B-130	P-091	B-130	C-200	B-062
	B-062	B-062	B-062	B-062	B-130
	B-001	M-051	M-051	B-130	M-051
	C-054	C-131	-	M-209	P-208
	C-106	M-092	-	-	-
APPLICATION *	Wk(3)**	Wk(2)	Wk(3)	Wk(4)	Wk(4)
**REPLICATION**	*n* = 6	*n* = 6	*n* = 6	*n* = 6	*n* = 6
**SUB-UNIT**	6 plants (18 leaves)/plot	6 plants (6 leaves)/plot	6 (whole) plants/plot	12 flowers (6 plants)/plot	6 (whole) plants/plot

* Wk = Weekly application, with number of applications in parenthesis. NB: Standards (and other treatments where labels existed) were applied according to commercial practice in the UK, where the maximum number of applications specified and used is provided in superscript. ** Except P-001 which was applied 4 times at 4–5 day intervals.

Statistical analysis was run in SPSS (v22, IBM, New York, U.S.; 2013) considering sampling dates independently and with treatment (*i.e.*, product) as the main and only factor. Data were checked for normality and homoscedasticity and transformed where possible to fit the assumptions of ANOVA and *post hoc* Tukey’s tests. Where data could not be made to conform to the requirements of parametric testing, analysis was run using a Kruskal Wallis test for main effects, with Bonferroni-Dunn tests used to conservatively explore pairwise differences. Further details of tests/transformations used are provided within Table 2, Table 3, Table 4, Table 5 and Table 6, where for ease of reference all data are displayed as means with standard errors.

## 3. Results

Results of trials are presented below by crop and target species.

### 3.1. Tomato: Trialuerodes vaporariorum

*Trialuerodes vaporariorum* populations established well, with both adults and larvae evenly infesting treatments at the start of the trial. Numbers increased in the control treatment up to six days after the second application (6DDA2), with reduced, but still high, numbers in the control six days after the third treatment application (6DDA3). For adults, a treatment effect was only observed 6DAA2, at which time all experimental pesticides, and the industry standard, had similarly reduced adult counts as compared to the control. The same pattern was observed for *T. vaporariorum* larvae 6DAA2, also being repeated at the final assessment 6DAA3.

Table 2Mean (±SE) number of *Trialuerodes vaporariorum* per plot (6 plants) under varying treatments pre- and post-treatment application. *n* = 6 for all means. *p* values display the significance of the overall treatment effect on any given sampling date. Means followed by a different letter within a row are significantly different at *p* < 0.05.

**Table:** 

Adults	Control	Chess WG	B-130	B-062	B-001	C-054	C-106	F_(6,35)_/χ^2^_(6)_; *p* value
D0	17.8 ± 3.5	17.3 ± 4.0	18.2 ± 4.3	12.0 ± 2.3	13.2 ± 2.9	13.3 ± 2.0	13.8 ± 2.6	(F)0.653; 0.687
6DAA1	16.2 ± 3.5	16.8 ± 3.8	11.5 ± 2.7	12.3 ± 1.8	19.7 ± 7.3	13.7 ± 1.3	10.8 ± 2.8	(χ^2^)2.507; 0.868
6DAA2	64.0 ± 9.7 ^a^	21.2 ± 2.9 ^b^	18.3 ± 6.6 ^b^	26.2 ± 9.6 ^b^	22.3 ± 4.8 ^b^	18.0 ± 6.3 ^b^	9.2 ± 2.8 ^b^	(F)7.107; <0.001
6DAA3	30.7 ± 13.9	17.5 ± 4.1	24.5 ± 5.2	18.7 ± 6.9	14.3 ± 4.1	14.7 ± 5.1	15.0 ± 5.9	(F)0.563; 0.756 *

* Data square root transformed prior to analysis.

**Table:** 

Larvae	Control	Chess WG	B-130	B-062	B-001	C-054	C-106	F_(6,35)_; *p* value
D0	71.2 ± 15.8	35.7 ± 12.9	93.5 ± 37.1	27.8 ± 10.7	44.3 ± 20.0	54.0 ± 14.1	36.8 ± 9.2	(F)1.475; 0.215 *
6DAA1	108.2 ± 25.2	50.7 ± 21.0	31.3 ± 8.3	28.7 ± 4.5	65.7 ± 22.8	72.2 ± 24.0	51.0 ± 20.2	(F)1.509; 0.204 *
6DAA2	201.8 ± 28.7 ^a^	35.0 ± 3.0 ^b^	47.7 ± 10.2 ^b^	54.3 ± 7.4 ^b^	44.5 ± 11.5 ^b^	59.0 ± 11.7 ^b^	38.3 ± 11.2 ^b^	(F)13.863; <0.001 *
6DAA3	165.2 ± 24.8 ^a^	22.7 ± 8.5 ^b^	36.0 ± 8.6 ^b^	67.7 ± 14.9 ^b^	39.7 ± 9.5 ^b^	66.0 ± 18.0 ^b^	56.2 ± 14.4 ^b^	(F)9.733; <0.001

* Data square root transformed prior to analysis.

### 3.2. Tomato: *Tetranychus urticae*

Adults and nymphs of *T. urticae* evenly infested treatments at the start of the trial, with experimental populations establishing well thereafter. For adults, a treatment effect was only observed 6DAA1, at which time the experimental pesticide C-131 had reduced numbers relative to the P-091 treatment. No other differences between treatments existed, though C-131 came close to reducing numbers as compared to the control 6DAA1 (*p* = 0.092), with no adults recovered from treated plants. Mean control counts were actually higher at this time than in the P-091 treatment, but notably more variable. For nymphs, the industry standard exerted a suppressive effect, this being statistically significant in comparison to the control 6DAA2. Notably, lowered counts against the control were also seen at this time for other experimental products, though control counts were highly variable and differences were not statistically significant.

### 3.3. Pepper: *Myzus persicae*

For *M. persicae*, an even infestation of treatments was achieved at the start of the trial, with numbers in the control treatment generally holding at a stable level thereafter. Treatment effects were observed 6DAA1 and 6DAA3, at which points the biopesticide B-130 had reduced numbers of *M. persicae* relative to the control, also outperforming other treatments, including the industry standard. No other differences between treatments existed, including for the industry standard as compared to the control. Consistently, albeit non-significantly, reduced aphid numbers were also observed in B-062 treated plots relative to the control.

**Table 3:** Mean (±SE) number of *Tetranychus urticae* per plot (6 plants) under varying treatments pre- and post-treatment application. *n* = 6 for all means. *p* values display the significance of the overall treatment effect on any given sampling date. Means followed by a different letter within a row are significantly different at *p* < 0.05.

Adults	Control	Borneo	P-091	B-062	M-051	C-131	M-092	F_(6,35)_/χ^2^_(6)_; *p* value
D0	9.8 ± 1.9	4.5 ± 1.2	16.0 ± 7.5	9.0 ± 3.1	10.2 ± 5.0	11.3 ± 4.0	10.7 ± 2.2	(F)0.853; 0.538 *
6DAA1	4.0 ± 1.9 ^ab^	2.0 ± 1.4 ^ab^	3.2 ± 0.9 ^a^	1.3 ± 0.3 ^ab^	2.0 ± 0.9 ^ab^	0.0 ± 0.0 ^b^	1.2 ± 0.5 ^ab^	(χ^2^)12.969; 0.044
6DAA2	18.0 ± 14.0	0.5 ± 0.5	0.8 ± 0.5	2.7 ± 1.1	0.3 ± 0.2	1.2 ± 0.5	2.8 ± 1.6	(χ^2^)9.218; 0.162
D0	56.2 ± 22.2	64.7 ± 15.4	82.3 ± 22.9	76.2 ± 15.8	84.8 ± 32.5	47.0 ± 7.8	62.0 ± 11.2	(F)0.502; 0.802
6DAA1	53.7 ± 20.3	16.5 ± 6.9	18.7 ± 4.8	17.7 ± 10.7	19.8 ± 6.7	6.8 ± 2.3	15.0 ± 6.2	(F)2.238; 0.062
6DAA2	42.0 ± 22.7 ^a^	1.3 ± 0.5 ^b^	4.7 ± 2.1 ^ab^	5.2 ± 1.7 ^ab^	6.5 ± 2.0 ^ab^	3.2 ± 1.9 ^ab^	5.3 ± 2.6 ^ab^	(χ^2^)13.609; 0.034

* Data square root transformed prior to analysis.

**Table 4:** Mean (±SE) number of *Myzus persicae* per plot (6 plants) under varying treatments pre- and post-treatment application. *n* = 6 for all means. Means are displayed with ± SEs. *p* values display the significance of the overall treatment effect on any given sampling date. Means followed by a different letter within a row are significantly different at *p* < 0.05.

Motiles	Control	Pyrethrum 5EC	B-130	B-062	M-051	F_(4,25)_; *p* value
D0	105.8 ± 30.9	94.5 ± 44.7	35.8 ± 15.0	35.0 ± 7.9	61.2 ± 16.7	1.156; 0.354 *
6DAA1	158.0 ± 41.8 ^a^	48.5 ± 13.7 ^ab^	14.4 ± 5.4 ^b^	52.7 ± 14.0 ^ab^	65.5 ± 25.9 ^ab^	3.441; 0.023 *
6DAA2	118.6 ± 28.6	97.8 ± 46.8	11.2 ± 4.4	68.5 ± 23.7	54.8 ± 19.2	2.441; 0.073 *
6DAA3	92.6 ± 13.8 ^a^	119.0 ± 39.4 ^a^	14.0 ± 3.8 ^b^	71.5 ± 13.5 ^ab^	116.8 ± 38.2 ^a^	5.390; 0.003 *

* Data square root transformed prior to analysis.

### 3.4. Pepper: Frankliniella occidentalis

Populations of *F. occidentalis* adults and nymphs established well, evenly infesting treatments following release. For adults, a treatment effect was observed 6DAA4, at which time the biopesticide B-062 had reduced pest numbers relative to the control. No other differences between treatments existed, including for the industry standard against the control. For nymphs, the industry standard did exert a significant effect, though with highest pest numbers observed under this treatment as compared to the control (6DAA3). Statistically significant reductions in nymph counts, compared to the control, were only observed for C-200, being evident 6DAA1, 6DAA2 and 6DAA4.

### 3.5. Pepper: Aulacorthum solani

Even establishment of *A. solani* populations was recorded across treatments prior to application of products. Numbers in control treatments initially increased between D0 and 6DAA1, though at this time the trial was infested by parasitoids. Thereafter aphid numbers declined rapidly, with counts in the control treatment being so low at the end of the trial that data collected 6DAA4 were not considered suitable for analysis. Six days after application 1 (6DAA1), when *A. solani* populations were growing in the control, reduced numbers of aphids, in comparison to the control, were observed under the industry standard treatment, where counts were also lower than in the P-208 treatment. This pattern continued 6DAA2, with numbers reduced in the B-062 treatment, relative to the P-208 treatment at this time. Reductions under the B-062 treatment were not significant in comparison to the control 6DAA2, though were more than an order of magnitude lower. By 6DAA3, differences between treatments could not be identified by (relatively conservative) *post-ho*c testing, though an overall treatment effect persisted.

Table 5Mean (±SE) number of *Frankliniella occidentalis* per plot (6 plants) under varying treatments pre- and post-treatment application. *n* = 6 for all means. *p* values display the significance of the overall treatment effect on any given sampling date. Means followed by a different letter within a row are significantly different at *p* < 0.05.

**Table:** 

Adults	Control	Calypso	C-200	B-062	B-130	M-209	F_(5,30)_; *p* value
D0	18.8 ± 3.2	12.8 ± 3.2	10.2 ± 2.5	12.5 ± 2.9	6.5 ± 1.4	13.5 ± 2.6	1.935; 0.118
6DAA1	24.8 ± 3.9	16.8 ± 3.4	17.0 ± 2.1	19.0 ± 3.8	23.2 ± 5.3	15.6 ± 5.3	0.776; 0.575 *
6DAA2	39.0 ± 4.9	32.3 ± 8.9	34.8 ± 5.5	26.8 ± 7.7	38.6 ± 8.7	17.7 ± 7.2	1.195; 0.335
6DAA3	41.5 ± 8.6	49.0 ± 17.8	30.0 ± 5.8	34.7 ± 8.4	44.5 ± 13.6	45.7 ± 6.3	0.521; 0.758 *
6DAA4	90.4 ± 9.1 ^ab^	116.5 ± 12.1 ^a^	87.4 ± 11.4 ^ab^	57.2 ± 6.1 ^b^	110.8 ± 13.2 ^a^	74.5 ± 12.0 ^ab^	4.160; 0.005

* Data square root transformed prior to analysis.

**Table:** 

Nymphs	Control	Calypso	C-200	B-062	B-130	M-209	F_(6,35)_; *p* value
D0	11.4 ± 4.7	14.2 ± 4.9	31.6 ± 6.3	24.2 ± 3.9	23.5 ± 7.4	11.3 ± 2.5	1.881; 0.127
6DAA1	28.2 ± 3.8 ^a^	14.7 ± 2.9 ^ab^	3.0 ± 1.7 ^b^	11.0 ± 5.4 ^ab^	10.8 ± 3.6 ^ab^	12.4 ± 4.8 ^ab^	3.246; 0.018 *
6DAA2	35.0 ± 4.6 ^ab^	34.3 ± 10.9 ^ab^	6.4 ± 1.9 ^ab^	16.8 ± 4.5 ^ab^	14.2 ± 5.5 ^ab^	17.1 ± 5.8 ^ab^	3.842; 0.008 *
6DAA3	10.5 ± 2.0 ^ab^	34.7 ± 7.6 ^ab^	7.4 ± 1.8 ^ab^	22.1 ± 5.1 ^ab^	15.2 ± 4.1 ^ab^	15.8 ± 4.9 ^ab^	4.196; 0.005
6DAA4	30.6 ± 5.5 ^ab^	44.8 ± 8.1 ^ab^	9.4 ± 1.0 ^ab^	35.5 ± 8.7 ^ab^	23.7 ± 4.0 ^ab^	27.5 ± 2.9 ^ab^	9.254; <0.001 **

* Data square root transformed prior to analysis; ** Data log transformed prior to analysis.

**Table 6:** Mean (±SE) number of *Aulacorthum solani* per plot (6 plants) under varying treatments pre- and post-treatment application. *n* = 6 for all means. *p* values display the significance of the overall treatment effect on any given sampling date. Means followed by a different letter within a row are significantly different at *p* < 0.05.

Motiles	Control	Chess WG	B-062	B-130	M-051	P-208	F_(5,30)_/χ^2^_(5)_; *p* value
D0	15.8 ± 2.7	20.8 ± 4.9	23.2 ± 6.4	15.2 ± 7.0	13.2 ± 3.8	14.3 ± 5.3	(F)0.829; 0.539 *
6DAA1	36.2 ± 9.8 ^a^	1.5 ± 0.6 ^b^	7.8 ± 4.7 ^ab^	19.8 ± 5.6 ^ab^	18.3 ± 4.4 ^ab^	35.2 ± 8.6 ^a^	(χ^2^)18.080; 0.003
6DAA2	7.8 ± 2.2 ^ac^	0.0 ± 0.0 ^b^	0.6 ± 0.5 ^bc^	1.7 ± 0.9 ^ab^	8.2 ± 3.4 ^ab^	20.7 ± 5.9 ^a^	(χ^2^)22.420; <0.001
6DAA3	3.0 ± 0.9	0.0 ± 0.0	0.8 ± 0.5	1.8 ± 1.3	6.2 ± 3.9	5.5 ± 2.0	(χ^2^)11.329; 0.045

* Data square root transformed prior to analysis.

## 4. Discussion

The aim of this study was to deliver applied research on high priority pests of glasshouse tomato and pepper crops in order to confirm relative efficacy of standard conventional chemical pesticides and support approval of “new” conventional and/or biopesticide products. In all experiments, at least one of the pesticide products tested provided control of the target pest on at least one post-treatment sampling date, relative to the control treatment. In some cases, the control provided by biopesticides was greater than that achieved through use of the industry standard, where the latter performed poorly as compared to the control in multiple trials. Thus, in addition to providing data in support of approval for effective pesticides in glasshouse tomato and pepper, this work also suggests that current use of some “standard” pesticides may need to be reviewed, particularly if these results are repeatable, or supported by industry trends for reduced or failing product efficacy.

Of those biopesticides that significantly suppressed pests on at least one sampling date in comparison to the control, B-130 demonstrated notable potential, being statistically effective against *T. vaporariorum* (adults and larvae) and *M. persicae*, and resulting in notable reductions in *A. solani* (to a maximum of 78%). Other botanical products also performed well when compared to controls, with B-062 reducing *A. solani* on peppers by more than an order of magnitude and statistically lowering adult and larval *T. vaporariorum* on tomato. It was ineffective against *M. persicae* and *F. occidentalis*, though did cause statistically non-significant reductions in average *T. urticae* counts on tomato of up to 88%. B-001 was also effective, reducing adult and larval *T. vaporariorum* counts on tomato in the only experiment that included this product.

Of the other pesticides tested in the current study, only experimental conventional chemical pesticides exerted any statistically significant effect on pest numbers, with microbials and physically acting products thus performing relatively poorly. M-092 showed some potential against both stages of *T. urticae*, though after adjusting *post-hoc p*-values for multiple comparisons pest reductions observed after treatment with M-092 were not statistically significant. Being reliant on living organisms, some degree of variability in the efficacy of microbials is perhaps to be expected. Such products often require relatively specific thresholds for temperature, moisture and UV exposure post-application, with sensitivity to these variables noted as a constraint to their effective use [22]. Nevertheless, many microbials may be highly selective, with application challenges arguably outweighed by their consequent compatibility with biocontrol [16]. Physically-acting, and in this case fatty-acid-based, pesticides should be less constrained by such “biological” requirements, though may be influenced by other “non-biological” factors. It is plausible in the current study, for example, that efficacy of these products was negatively affected by relatively high water hardness at the experimental site, which may have resulted in the “active ingredient” precipitating out of solution (D. George, personal observation).

The efficacy of industry standards in the current study varied greatly, with some being entirely ineffective against target pests. This was particularly so for Calypso when used against *F. occidentalis* in peppers and Pyrethrum 5EC when deployed against *M. persicae* in peppers. The latter of these pesticides is itself a plant-based formulation, though one widely adopted and with a long history of use. This prolonged use may explain the lack of efficacy of Pyrethrum 5EC in the current study, where it is possible that prior exposure of the *M. persicae* used to pyrethrins/pyrethroids had led to evolution of resistance in the experimental pest population to this group of actives. Pesticide resistance is widely reported in *M. persicae*, being first documented in 1955 (to organophosphates) [23]. *Myzus persicae* has since developed resistance to numerous actives, including pyrethroids, and is currently considered one of the world’s most strongly resistant pest species [8]. *Frankliniella occidentalis* are also noted as readily developing resistance [9], though conversely Calypso is an entirely synthetic product with thiacloprid (a neonicotinoid) as its active ingredient. Nevertheless, in a study investigating the contact toxicity of 36 pesticides to *F. occidentalis*, Shan *et al.* [24] reported thiacloprid to be the least effective tested. Using the glass-vial method, the LC_50_ of thiacloprid to larval *F. occidentalis* exceeded 40,000 mg/L in this work, with that of the most effective product (Phoxim) being 0.003 mg/L for comparison. This apparent lack of toxicity of thiacloprid to *F. occidentalis* is supported by the results of the current study. For species such as *M. persicae* and *F. occidentalis* that are clearly sometimes difficult to control using existing industry standards, studies investigating alternative control options are critical. The current study supports that B-130 could be of use for *M. persicae* and that C-200 holds promise to target *F. occidentalis* nymphs. C-106 was also effective in the current study (against *T. vaporariorum*), with C-131 causing large, albeit statistically non-significant, reductions in average *T. urticae* counts.

Even where current industry standards performed well, there is still a need to identify biopestiocides and alternative conventional chemical pesticides to safeguard against product failures and/or withdrawals of existing pesticides, and better align current and future glasshouse production with EU legislation. According to a recent UK report by farm business consultants Andersons, numerous existing conventional chemical pesticides are being, or are expected to be lost as a result of EC 1107/2009 [11], with increased pressure being placed on growers to adopt integrated pest management approaches via the Sustainable Use Directive (Directive 2009/128/EC). One advantage of many biopesticides is that they often perform better than conventional chemical pesticides in an integrated approach; displaying short residual activities that lend well to combined use with subsequent biological control releases [16]. Complex chemistries and potentially multi-facetted modes of action (e.g., certain botanicals) may also provide better long-term resistance management where such products are used [13,14], though immunity of biopesticides to pest resistance should not be assumed [14]. Microbial/physically-acting products are expected to be similarly robust in this regard, though it deserves note that resistance to Bt has already been reported in multiple pest species [25].

Though use of biopesticides requires a more considered approach to pest management than treatment with conventional pesticides, requiring extra care in product handling, application and treatment timings [19], the results presented support that these products, and especially botanical biopesticides, hold promise in glasshouse production. Guidance to optimise the uptake of biopesticides in the UK, and ensure that potential limitations are alleviated by broader benefits, is being well supported through levy board technology transfer.

## 5. Conclusions

The work presented supports that existing industry pesticides may be variable in efficacy, supporting the need to investigate potential alternative products. The current project has identified a number of experimental conventional chemical and botanical products that show significant potential to contribute to tomato and bell pepper production under glasshouse conditions, the latter being particularly attractive in the current legislative climate where existing conventional chemistries are under threat and integrated pest management is prioritised. Work is on-going to ensure that the results of this study translate into increased pesticide product availability for the glasshouse vegetable industry in the UK.
